# Effect of Structural Design on the Pore Structure, Water Resistance, and Mechanical Properties of Engineered Wood/Bamboo Laminated Composites

**DOI:** 10.3390/polym14245386

**Published:** 2022-12-09

**Authors:** Xuguang Zhu, Xiaoyan Li, Zhiyuan Zhang, Lin Cheng, Jue Wu, Luming Li, Zhenhua Zhang, Qingyuan Liu, Pu Zhao, Fei Rao

**Affiliations:** 1 School of Art and Design, Zhejiang Sci-Tech University, Hangzhou 310018, China; 2 China National Bamboo Research Center, Wenyi Road 310, Hangzhou 310012, China; 3 Nanjing Pukou Cultural Tourism Operation Management Co., Ltd., Xigeng Lianxiang Branch, Nanjing 211800, China; 4 Guangxi Key Laboratory of Superior Timber Trees Resource Cultivation, Forestry Research Institute of Guangxi Zhuang Autonomous Region, Nanning 530002, China; 5 College of Chemistry and Materials Engineering, Zhejiang A&F University, Hangzhou 311300, China

**Keywords:** wood/bamboo laminated composite, structural design, density, pore structure, water resistance, mechanical properties

## Abstract

An important principle in rational manufacturing design is matching the properties of composites to their intended uses. Herein, six laminated composites (LCs) were manufactured using fibrous moso bamboo and poplar veneer units, and their pore structure, water resistance, and mechanical properties were evaluated. The LC density (640–1290 kg/m^3^) increased significantly with increasing bamboo veneer unit content. The LC surface texture and roughness depended on the density and type of surface layer. With increasing LC density, the water absorption rate (WAR), width swelling rate (WSR), and thickness swelling rate (TSR) decreased exponentially and the mechanical properties increased linearly. This behavior was closely related to the changes in pore structure caused by density. Notably, the water resistance and mechanical properties of the LCs with densities higher than 910 kg/m^3^ were superior to the highest levels specified in GB/T 20241–2006 for ‘‘laminated veneer lumber’’ and GB/T 30364–2013 for “bamboo scrimber flooring”. Thus, these engineered materials are promising for outdoor structures and flooring.

## 1. Introduction

The construction industry has a considerable impact on the environment [1], accounting for 40% of global energy and 36% of energy-related carbon emissions in industrialized countries [2]. Wood resources have unique advantages, such as the ability to sequester carbon and renewability. Moreover, compared with plastics, cement, and steel, wood has wider applicability in the field of architectural engineering [3]. Although China has abundant bamboo and poplar resources, the uneven structure, anisotropy, and poor durability of these materials has greatly limited their application in engineered structures. An effective way to overcome these shortcomings and efficiently utilize wood resources is through the design of bamboo and wood veneer stratification units with the directional reorganization of fibers. This approach can be used to prepare bamboo and wood laminated composites (LCs) with uniform structure, excellent performance, and strong durability [3].

Scrimber is a type of composite biomass material formed by the directional recombination and gluing of raw biomass materials such as plantation wood, bamboo, and sandy shrubs. Since 2014, the Wood Industry Research Institute of the Chinese Academy of Forestry has successively developed novel high-performance wood and bamboo scrimber with a modulus of rupture (MOR) of more than 364 and 200 MPa, respectively, and a thickness swelling rate (TSR) of less than 0.6% and 6%, respectively. Such performance characteristics indicate that these products are widely applicable as building and flooring materials.

Generally, owing to the characteristics of wood, 4–8 mm thick veneers can be obtained by generating a series of linear cracks along the grain direction using a crushing machine with different types of gears. Subsequently, the veneers can be directionally fluffed and separated into fibrous wood veneer units composed of wood fiber bundles of uniform thickness. This fluffing process can significantly improve the dimensional stability of wood scrimber, decreasing the TSR by approximately 74%, without significantly impacting the mechanical properties [4]. In addition, as the wood veneer unit thickness increases, the mechanical properties of wood scrimber gradually deteriorate, whereas the dimensional stability improves [5].

Based on the manufacturing method for wood scrimber, the successful development of bamboo scrimber can fully utilize China’s rich bamboo resources. Bamboo is first truncated in the transverse direction and split longitudinally into two semicircular tubes, which are then mechanically fluffed and flattened to form fibrous bamboo veneer units with reticular structure. As this process does not require the removal of the outer and inner walls of bamboo culm, the bamboo utilization rate increases from 50–55% to 92–95% [6]. The siliceous and waxy layers of bamboo culm do not readily bond with resin and are responsible for most shedding and destruction after mechanical treatment. When bamboo fibers, vessels, and basic tissue are separated, the maximum fiber strength is retained and stress on the inner wall of the bamboo tube is reduced. Moreover, the permeability of bamboo to resins is significantly improved, which provides a basis for high-performance bamboo lamination. The physical and mechanical properties of novel bamboo scrimber prepared with fibrous bamboo veneer are significantly better than those of traditional bamboo scrimber prepared with bamboo bundles [7].

However, compared with wood scrimber, bamboo scrimber has a high density, excessive mechanical properties, and low dimensional stability and durability. To realize balanced physical and mechanical properties, wood/bamboo LCs can be produced using fibrous bamboo and wood veneer units. Based on our previous research [8,9], this bamboo and wood LCs approach allows for the comprehensive, high-quality, and efficient utilization of wood and bamboo resources. Chen et al. studied the preparation of bamboo/wood LCs and found that the optimal process at a bamboo/wood ratio of 3:2 and an adhesive solid content of 25% gave a density of 1100 kg/m^3^ [10]. The modulus of elasticity (MOE) and MOR of a poplar/bamboo bundle LC with a density of 760 kg/m^3^ were higher than those of a poplar veneer LC. Based on the MOE and shear strength (SS), a structural LC with a density of 760 kg/m^3^ exhibited a strength value superior to the 140E level and 65V-55H grade, making it suitable for engineered laminated veneer lumber, as specified in GB/T 20241–2006 for “laminated veneer lumber” [11]. Compared with other fibrous materials, bamboo fiber bundles separated by thermal–mechanical treatment have advantageous tensile properties, which can enhance the MOR and MOE of poplar/bamboo fiber LCs. In bamboo bundle/wood veneer LCs with different structures (density: 660–886 kg/m^3^), the mechanical properties improve as the bamboo bundle content increases. In particular, when the bamboo bundles form the surface layer of the laminated structure (BBPPPBB), the MOE, MOR, and SS perpendicular to the glue line direction can reach 243.4 MPa, 22.8 GPa, and 16.7 MPa, respectively [12]. Wood/bamboo LCs made from fibrous bamboo (*Neosinocalamus affinis*) and poplar (*Populus* ssp.) veneer units have excellent water resistance and mechanical properties, indicating that they are promising materials for structures and flooring, particularly for outdoor platforms and landing stages [9]. Therefore, wood/bamboo LCs are an innovative development in wood and bamboo scrimber, and we propose the hypothesis that LCs with different densities can be obtained through different structural design using poplar and bamboo veneer units. The purpose of the study is to explore a simple new method to adjust and control the physical and mechanical properties of wood- and bamboo-based composite materials, to achieve a reasonable match between properties and use.

In this study, six LCs based on three different layup structures were designed using fibrous moso bamboo and poplar veneer units to evaluate the effect of structural design on the water resistance, mechanical properties, and pore structure of engineered wood/bamboo LCs.

## 2. Materials and Methods

### 2.1. Raw Materials

Four-year-old moso bamboo (*Phyllostachys edulis*) was obtained from Anji County, Zhejiang Province, China. Poplar wood (*Populus* ssp.), 4–5 years of age, was obtained from Shandong Province, China. Low-molecular-weight phenol-formaldehyde (PF) resin with a solid content of 47.9 wt%, a viscosity of 35 cps, and pH 10–11 was supplied by Beijing Dynea Chemical Industry Co. Ltd. (Beijing, China).

### 2.2. Structural Design and Manufacture of Wood/Bamboo LCs

**Figure 1:**
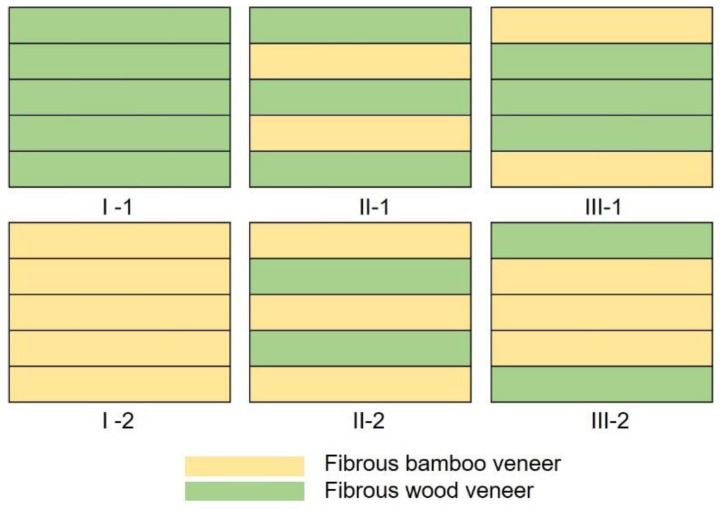
Layup designs of wood/bamboo LC panels.

Poplar wood veneers with a length, width, and thickness of 2000 mm, 20 mm, and 6 mm, respectively, were obtained through rotary cutting of poplar logs. Bamboo culm was processed into two 2000 mm long semicircular bamboo tubes by transverse truncation and longitudinal splitting. The semicircular bamboo tubes and wood veneers were then mechanically decomposed into continuous-oriented fluffed veneers using a multifunctional crushing machine. Fibrous bamboo and poplar veneer units (thickness: 6.7 ± 0.2 mm and 5.5 ± 0.1 mm, respectively) were prepared according to previously published methods [8]. The dimension of fibrous poplar and bamboo veneer was 2000 mm× 220 mm × 6.7 mm and 2000 mm × 28 mm× 5.5 mm, respectively. The fibrous veneer units were impregnated with a PF resin solution to achieve a resin solid content of 17 wt% and then dried naturally to a moisture content of 10–12%. Six wood/bamboo LCs with three layup structures (named I, II, and III) and two compositions (named 1 and 2) were manufactured (Figure 1). Each LC consisted of five veneer unit layers laminated along the grain direction in a 450 mm × 160 mm × 15 mm mold by hot pressing at 150 °C for 30 min [8]. The pressure was kept at 3.5–7.0 MPa to cure the resin. After two weeks of storage in indoor conditions, test samples of different dimensions were prepared by sanding and cutting slabs.

### 2.3. Characterization

The densities of the LC samples were tested according to the GB/T 17657–2013 standard [13]. The surfaces and cross-sections of the LC samples were examined using a digital microscopic system (VHX-6000, KEYENCE, Osaka, Japan). The pore sizes, pore size distributions, and porosities of the LC samples were measured quantitatively using a mercury intrusion porosimeter (MIP; AutoPore IV9500, Micromeritics, Norcross, GA, USA). The pressure range for intrusion was 0–227 MPa. Bending and shear tests were performed parallel to the face grains using a universal testing machine (MDW-W50, Jinan Time Testing Instrument Co., Ltd., Jinan, China) in the three-point bending mode according to GB/T 17657–2013 [13] and GB/T 20241–2006 [11], respectively. Following test procedure GB/T 30364–2013 [14], a 28 h treatment was used to determine the water absorption rate (WAR), width swelling rate (WSR), and TSR of the LC samples. The sample dimensions and test details are summarized in Table 1.

### 2.4. Statistical Analysis

Statistical analysis was performed using Duncan’s multiple range test followed by analysis of variance (ANOVA) at a significance level of 5%. All analyses were performed using the SPSS software (Version 25, IBM, Armonk, NY, USA).

## 3. Results and Discussion

### 3.1. Density

**Figure 2:**
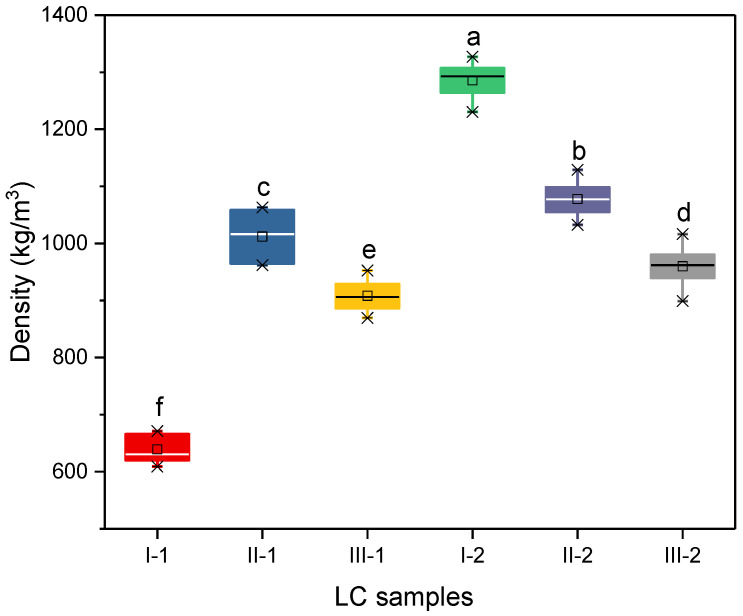
Densities of the LC samples. Different letters indicate significant difference at *p* < 0.05.

The densities of the six LCs ranged from 640 (I-1) to 1290 kg/m^3^ (I-2) (Figure 2), and an ANOVA revealed significant differences in the densities of all the samples. As shown in Figure 2, the densities of the other samples could be arranged in the following order: II-2 (1080 kg/m^3^) > I-2 (1010 kg/m^3^) > III-2 (960 kg/m^3^) > III-1 (910 kg/m^3^). This variation is due to the density differences between poplar (480 kg/m^3^) [15] and moso bamboo (680 kg/m^3^) [6] being amplified when their fibrous veneers were manufactured into LC panels of the same thickness with the same number of layers. The density of the LCs increased significantly as the bamboo veneer unit content increased, because the weight of single-layer bamboo veneer was greater than that of poplar veneer. In addition, the water uptake of poplar (123%) [15] is more than twice that of moso bamboo (57%) [8], which could lead to obvious differences in the resin solid content of their veneers after PF resin impregnation. The range of LC densities could promote the application of these materials in a variety of fields and scenarios.

### 3.2. Surface and Cross-Section

**Figure 3:**
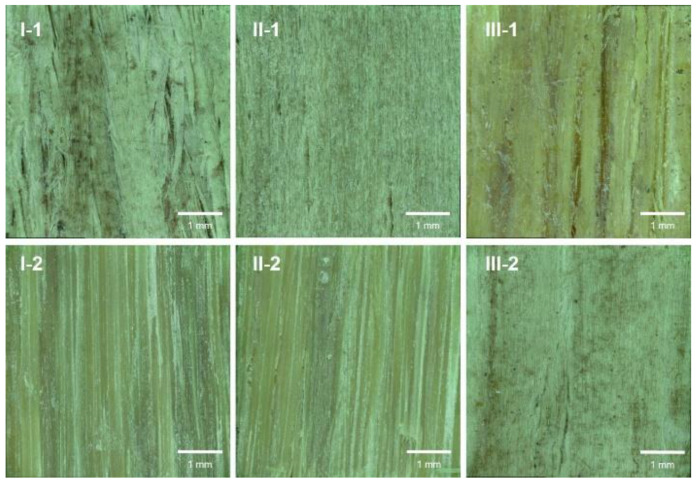
Surface textures of the LC samples.

**Figure 4:**
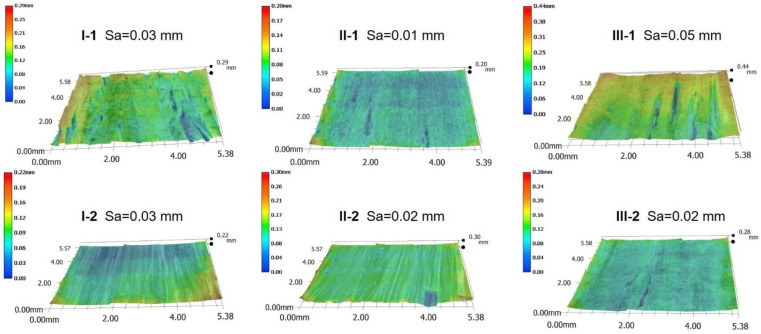
3D surface images of the LC samples. Sa = arithmetic average height (area).

Surface texture is a crucial factor for wooden materials when used by consumers in decoration applications. The LC surface texture depended on the type of surface veneer and the density. I-1, II-1, and III-2 exhibited a wood-like texture with poplar as face layer (Figure 3), with the amount of surface cracks (Figure 3) and roughness (Figure 4) decreasing as the density increased (Figure 2). In the same volume of mold, the higher the compression degree of high-density LCs, the lower the surface roughness under high pressure. Similarly, denser I-2 and II-2 exhibited a more closed surface with less surface cracks due to missing material and lower surface roughness than III-1 (Figure 3 and Figure 4).

**Figure 5:**
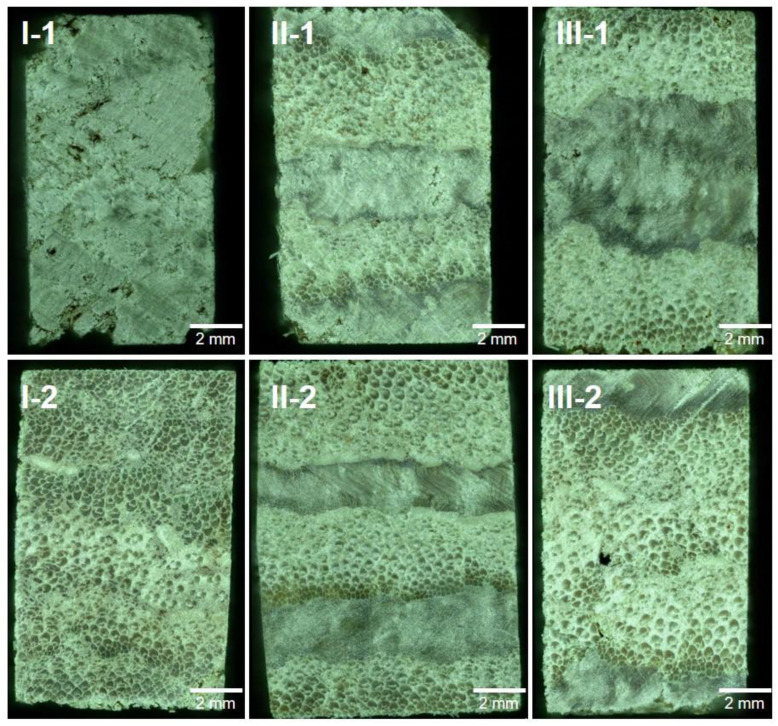
Optical microscopy images of the LC cross-sections.

The layup structures and compositions of the LCs could be visually observed in cross-sectional images (Figure 5). We first observe the cross section, and then select a representative cross section with an area of about 5 mm × 5 mm as the test area. Mechanical meshing between the veneer layers to form an interlocking bonding interphase is the basis for the physical and mechanical properties of the LCs [16]. In low-density I-1, the presence of more macropores was unfavorable for interphase bonding. In medium-density II-1 and III-2, most pores were located in or between the bamboo veneers, mainly because the stiff fiber caps of the bamboo vascular bundles were more difficult to compress. No macroscopic pores were observed in the cross-sections of high-density I-2 and II-2, indicating that these samples had better moisture-induced dimensional stability. No macroscopic pores were observed in the middle bamboo layer of II-2. This was because the density of II-2 (1080 kg/m^3^) was greater than II-1 (1010 kg/m^3^) and III-2 (960 kg/m^3^).

### 3.3. Pore Structure

The MIP results for the LC samples are summarized in Table 2. The highest and lowest porosities were observed for the LCs with structure I (9.8% for I-2 and 39.3% for I-1), whereas the porosities of the other samples were distributed between 30.6% and 35.1%. There was a negative correlation between the porosity and density of the LCs. The same trend was observed for the total intrusion volume and bulk density, indicating that the internal pore volumes of the samples with structures II and III were between those of I-1 and I-2. The average pore diameter of low-density I-1 was one order of magnitude higher than those of the other LCs, which was due to the presence of numerous macropores (Figure 5) resulting from the low compression ratio of this sample. The average pore diameters of II-1 and III-1 were higher than those of II-2 and III-2 because the former had lower bamboo/wood veneer ratios than the latter. The pressure on III-2 was mostly absorbed by the soft poplar veneer surface layer, which made it difficult to effectively densify the three core layers of harder bamboo veneer (Figure 5). Consequently, the average pore diameter of III-2 was greater than that of II-2. The opposite trend was observed for II-1 and II-2.

**Figure 6:**
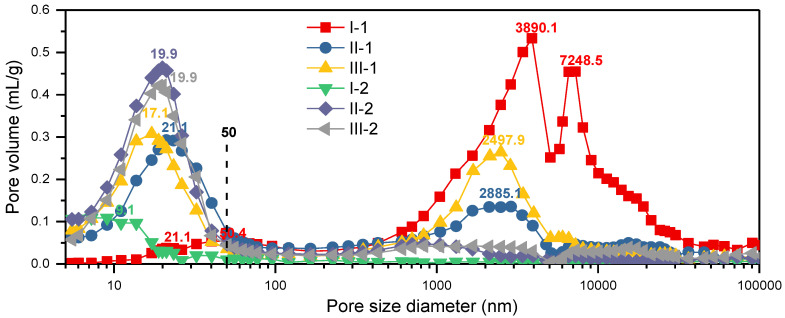
Pore size distributions of the LC samples.

The log differential intrusion as a function of pore diameter (Figure 6) reflected the pore size distribution of each LC. The bimodal pore size distribution of I-1, II-1, and III-1 was macroscopic pores created by lamination and micropores of bamboo and poplar itself. The II-2 and III-2 with higher density had less macroscopic pores (>50 nm). The I-2 with the highest density exhibited that the pore size distribution consists of mostly below 50 nm, which was consistent with our previous research [8]. In I-1, the pores were mainly distributed in the range of 3–8 µm, with some pores also observed at 50.4 and 21.1 nm. In I-2, the pores were mainly distributed around 9.1 nm, corresponding to the cell cavity pores of bamboo fiber cells and compressed parenchyma cells, as well as pits not filled with resin [8]. Owing to the differences in the microstructures of moso bamboo and poplar, the pore structure and quality of the LCs differ considerably. For the samples with structures II and III, the differences in the pore size distributions mainly depend on the bamboo/wood veneer unit ratio and the laminated structure.

### 3.4. Water Resistance

**Figure 7:**
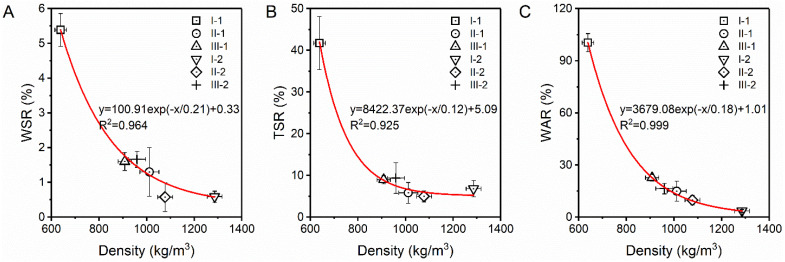
Water resistance of the LC samples: (**A**) WSR, (**B**) TSR, and (**C**) WAR.

The water resistance reflects internal stress release in the LCs under extreme hydrothermal conditions. The WSR, TSR, and WAR of the LCs decreased exponentially with increasing density, and the coefficients of determination (R^2^) were all greater than 0.92 (Figure 7). Low-density I-1 had the highest WSR, TSR, and WAR values, indicating the worst water resistance, which is due to the large number of macropores in this sample. The WSR values of I-2 and II-2 were significantly lower than those of the other samples. The TSR values of samples with densities higher than 1000 kg/m^3^ (I-2, II-1, and II-2) were significantly lower than those of the lower density samples (I-1, III-1, and III-2). The low-molecular-weight PF resin penetrates cell walls under high pressure and is then heat cured to form a three-dimensional interpenetrating polymer network (IPN) [8,16]. This hydrophobic resin film effectively prevents the hydroxyl groups of the veneer units from interacting with water molecules to cause irreversible swelling. The occurrence of spring back under drastic conditions is limited by the mechanical interlocking structure between the cells through an anchor, hook, or nail-like cured resin on the interior and exterior of the cell walls [16]. The denser pore structures (Figure 5) and more severe cell lumen deformation (Figure 6) of the high-density composites after the hot-pressing densification process were more conducive to IPN formation, resulting in low WSR and TSR values. Furthermore, the TSR value of I-2 with a density of 1290 kg/m^3^ was higher than those of II-1 and II-2 with densities of 1010–1080 kg/m^3^. The stiff bamboo cell lumen in I-2 may store a huge transversal compressive stress under high pressure, whereas the softer poplar cells in II-1 and II-2 absorb part of the stress owing to their preferential plasticization. Notably, II-1, III-1, I-2, and III-2 meet the requirements of salable products for outdoor use (WSR ≤ 4%, TSR ≤ 10%), whereas II-2 meets the requirements of high-class products for outdoor use (WSR ≤ 3%, TSR ≤ 5%), as specified in the GB/T 30364–2013 standard for “bamboo scrimber flooring” [14]. The low dimensional stability of I-2 was due to the huge stress stored in cells during compression, and the stress rebound was easy to occur under hydrothermal conditions [7]. An exponential relationship was observed between the WAR and LC density (Figure 7A), which indicates that the pore structure is the primary factor affecting water absorption.

### 3.5. Mechanical Properties

**Figure 8:**
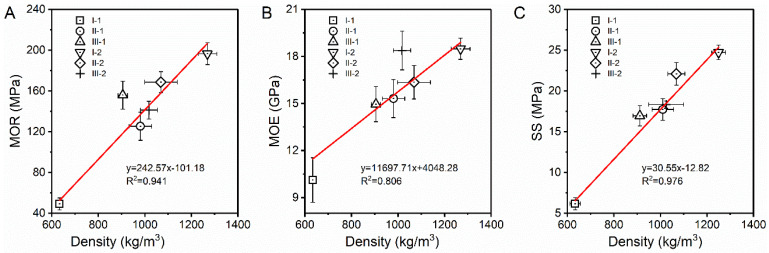
Mechanical properties of the LC samples: (**A**) MOR, (**B**) MOE, and (**C**) SS.

A good linear relationship was found between the LC density and the mechanical properties (MOR, MOE, and SS), with R^2^ values greater than 0.81 (Figure 8). I-1 had the lowest mechanical indices owing to its low density. The MOR of the other samples increased in the following order: II-1 (125.3 MPa) < III-2 (141.3 MPa) < III-1 (155.9 MPa) < I-2 (196.6 MPa). The MOR value of III-1 with a density of 910 kg/m^3^ was higher than those of II-1 with a density of 980 kg/m^3^ and III-2 with a density of 1020 kg/m^3^ but lower than that of I-2 with a density of 1290 kg/m^3^. These trends indicate that the bamboo veneer units could bear more tensile stress as the surface layer and more bending stress as the core layer than the poplar veneer units in the LCs. The MOE values of I-2 and III-2 (>18.0 GPa) were significantly higher than those of the other samples, indicating that the LCs containing a core layer consisting of three continuous bamboo veneer units were more rigid and not prone to bending deformation. The main reasons for the excellent mechanical properties of I-2 are that the densification process promotes the significant increase of fiber content, and the resin solidified between cells transfers the load to the fiber efficiently [16]. The bending properties of I-2 and III-2 correspond to a strength index of the highest level (180 E), making these LCs superior products for engineered laminated veneer lumber, as specified in GB/T 20241–2006 [11] for ‘‘laminated veneer lumber.’’ The corresponding strength values of I-2 and III-2 were 18E-135f (MOE ≥ 18 GPa, MOR ≥ 135 MPa), which conform to the LY/T 3194-2020 [17] standard for ‘‘structural bamboo scrimber’’, indicating that both these LCs are suitable for structural applications. The SS values of the LCs increased linearly with increasing density, which may be due to the veneer units in the high-density LCs being more tightly fixed by the resin, resulting in a higher load transfer efficiency and stronger resistance to interlaminar damage. The SS values of all the samples, except I-1, met the requirement for high-class outdoor-use bamboo scrimber flooring products (≥12 MPa), as specified in GB/T 30364–2013 [14]. LC samples began to break in micro-cracks at the interface of the inner and outer wall layers, then at the tension faces of the samples at mid-span.

## 4. Conclusions

In this study, six wood/bamboo LCs with three layup structures were prepared using fibrous moso bamboo and poplar veneer units as raw materials. For each LC, the pore structure, water resistance, and mechanical properties were evaluated. The density of the LCs increased significantly as the bamboo veneer unit content increased, ranging from 640 kg/m^3^ (I-1) to 1290 kg/m^3^ (I-2). The water resistance and mechanical properties of the materials, which increased exponentially and linearly, respectively, with increasing density, were closely related to the density-caused changes in pore structure. Using poplar and bamboo as raw materials, LCs prepared by structural design showed a wide range of performance. The physicomechanical properties of all the LC samples, except I-1, exceeded the specifications of GB/T 20241–2006 and GB/T 30364–2013, indicated that these composites with excellent comprehensive performance have considerable application potential in outdoor structures and flooring. Our research on the reasonable structural design of LCs provides beneficial guidelines for correlating the performance and use of wood- and bamboo-based composites.

## Figures and Tables

**Table 1:** Experimental test details for LC samples.

Property	Test Method	N	Sample Size (mm^3^)	Span (mm)	Loading Rate (mm/min)	Standard
Density	Mass/volume method	8	50 × 50 × 14	—	—	GB/T 17657–2013
Longitudinal bending properties	Three-point bending	8	330 × 20 × 14	280	10	
Longitudinal shear strength	Short-beam shear	8	78 × 40 × 14	52	5	GB/T 20241–2006
Water resistance	28 h cycle treatment	8	50 × 50 × 14	—	—	GB/T 30364–2013
Pore structure	Mercury intrusion method	3	1.5 × 1.5 × 0.9	—	—	—

**Table 2:** Mercury intrusion porosimeter (MIP) results for the LC samples.

Sample	Total Intrusion Volume (mL/g)	Average Pore Diameter (nm)	Bulk Density (g/mL)	Porosity (%)
I-1	0.5	363.4	0.7	39.3
II-1	0.4	34.6	0.9	31.4
III-1	0.4	32.0	0.9	35.1
I-2	0.1	13.6	1.2	9.8
II-2	0.3	19.7	1.0	30.6
III-2	0.3	22.6	1.0	30.6

## Data Availability

The data presented in this study are available on request from the corresponding author.
